# Diagnosing adult and pediatric extrapulmonary tuberculosis by MPT64 antigen detection with immunohistochemistry and immunocytochemistry using reproduced polyclonal antibodies

**DOI:** 10.1002/2056-4538.12373

**Published:** 2024-04-04

**Authors:** Ole Magnus Bjørgaas Helle, Mala Kanthali, Sheeba Ishtiaq, Atiqa Ambreen, Manju Raj Purohit, Tehmina Mustafa

**Affiliations:** ^1^ Department of Thoracic Medicine Haukeland University Hospital Bergen Norway; ^2^ Centre for International Health, Department of Global Public Health and Primary Care University of Bergen Bergen Norway; ^3^ Department of Pathology R.D. Gardi Medical College Ujjain India; ^4^ Department of Histopathology Gulab Devi Hospital Lahore Pakistan; ^5^ Department of Microbiology Gulab Devi Hospital Lahore Pakistan; ^6^ Department of Public Health Sciences Karolinska Institutet Stockholm Sweden

**Keywords:** extrapulmonary tuberculosis, childhood tuberculosis, antigen detection test, immunohistochemistry, immunocytochemistry, composite reference standard

## Abstract

Diagnosing extrapulmonary tuberculosis (EPTB) is challenging. Immunohistochemistry or immunocytochemistry has been used to diagnose tuberculosis (TB) by detection of MPT64 antigen from various extrapulmonary specimens and has shown good diagnostic performance in our previous studies. The test can distinguish between disease caused by *Mycobacterium tuberculosis* (Mtb) complex and nontuberculous mycobacteria and can be applied on formalin‐fixed paraffin‐embedded tissue. As the antibodies previously used were in limited supply, a new batch of polyclonal antibodies was developed for scale‐up and evaluated for the first time in this study. Our aim was to assess the diagnostic accuracy of the MPT64 test with reproduced antibodies in the high burden settings of Pakistan and India. Patients were enrolled prospectively. Samples from suspected sites of infection were collected and subjected to histopathologic and/or cytologic evaluation, routine TB diagnostics, GeneXpert MTB/RIF (Xpert), and the MPT64 antigen detection test. Patients were followed until the end of treatment. Based on a composite reference standard (CRS), 556 patients were categorized as TB cases and 175 as non‐TB cases. The MPT64 test performed well on biopsies with a sensitivity and specificity of 94% and 75%, respectively, against a CRS. For cytology samples, the sensitivity was low (36%), whereas the specificity was 81%. Overall, the MPT64 test showed higher sensitivity (73%) than Xpert (38%) and Mtb culture (33%). The test performed equally well in adults and children. We found an additive diagnostic value of the MPT64 test in conjunction with histology and molecular tests, increasing the yield for EPTB. In conclusion, immunochemical staining with MPT64 antibodies improves the diagnosis of EPTB in high burden settings and could be a valuable addition to routine diagnostics.

## Introduction

Diagnosing tuberculosis (TB) has been identified as the weakest link in the TB cascade of care [1], representing a major barrier for achieving the UN sustainable development goals of eradicating TB [2]. Diagnostic challenges are particularly high for extrapulmonary TB (EPTB) due to the varied clinical presentations, the paucibacillary nature of the disease, and the difficulty in obtaining appropriate specimens for analysis [3]. The performance of routine TB diagnostics such as culture and molecular tests is poor in cell smears and biopsies, which are important samples for laboratory diagnosis of various forms of EPTB. Even the WHO recommended GeneXpert MTB/RIF (Xpert) assay (Cepheid, Sunnyvale, CA, USA) has a sensitivity ranging between 50% and 82% for EPTB depending on the site of disease and type of specimen, using culture as reference standard [4]. In addition, not all sample types can be used in the Xpert instruments, particularly formalin‐fixed paraffin‐embedded tissue, for which a simple processing method for molecular testing is not widely available [5]. Therefore, EPTB is often diagnosed based on cytology or histopathology alone, with an emphasis on features of granulomatous inflammation. However, these features may also be present in conditions and diseases other than TB [6], and many TB patients may have atypical histopathologic or cytologic changes, making diagnosis difficult [7, 8, 9]. Furthermore, the distinction between disease caused by *Mycobacterium tuberculosis* (Mtb) and nontuberculous mycobacteria (NTM) is not possible based on histopathology or cytology. Suboptimal EPTB diagnosis leads to widespread empirical treatment, resulting in either over‐ or under‐treatment. This causes unnecessary patient costs and morbidity, and increases the risk of developing drug resistance to key anti‐TB chemotherapy.

To address the need for improved diagnosis of EPTB in cell smears and biopsies, an antigen detection test has been developed for detection of the Mtb specific antigen MPT64 by immunohistochemistry (IHC) or immunocytochemistry (ICC). This antigen is a protein secreted by the Mtb complex and cannot be detected in NTM infections [10, 11]. Immunohistochemical and immunocytochemical staining with MPT64 antibodies has shown promising diagnostic performance in a variety of extrapulmonary specimens in case–control laboratory studies and has been validated in cohort‐based diagnostic accuracy studies [12, 13, 14, 15, 16, 17]. The test method is rapid and robust, and its relative simplicity makes it suitable for implementation in low‐resource settings with modest laboratory facilities. Recent results from validation of the MPT64 test in routine diagnostics in the resource‐limited and high TB burden areas of Zanzibar and Tanzania show sensitivity and specificity of 69–92% and 78–95%, respectively, against a composite reference standard (CRS) [18, 19, 20]. However, the antibodies evaluated in previous studies were in limited supply. As sufficient supply is a prerequisite for a test to be used in routine diagnostics, a strategy has been developed for producing larger volumes of the MPT64 polyclonal antibodies, which can facilitate the use of this test on a large scale [21]. The aim of this study was to assess the performance of the MPT64 test with the reproduced polyclonal antibodies for the diagnosis of EPTB in the high TB burden settings of Pakistan and India, and to compare the results with routine diagnostics including Xpert.

## Materials and methods

### Ethical considerations

The study was approved by the Regional Committee for Medical Research Ethics in Norway, REK Helse‐Vest (2014/46/REK vest), the Ethical Committee in India (IEC 10/2018), and the Institutional Review Board, Al‐Aleem Medical College, Lahore, Pakistan (GDEC/234/18). Written informed consent was obtained from all participants. None of the invasive procedures was performed for research purposes only.

### Sample inclusion

**Figure 1:**
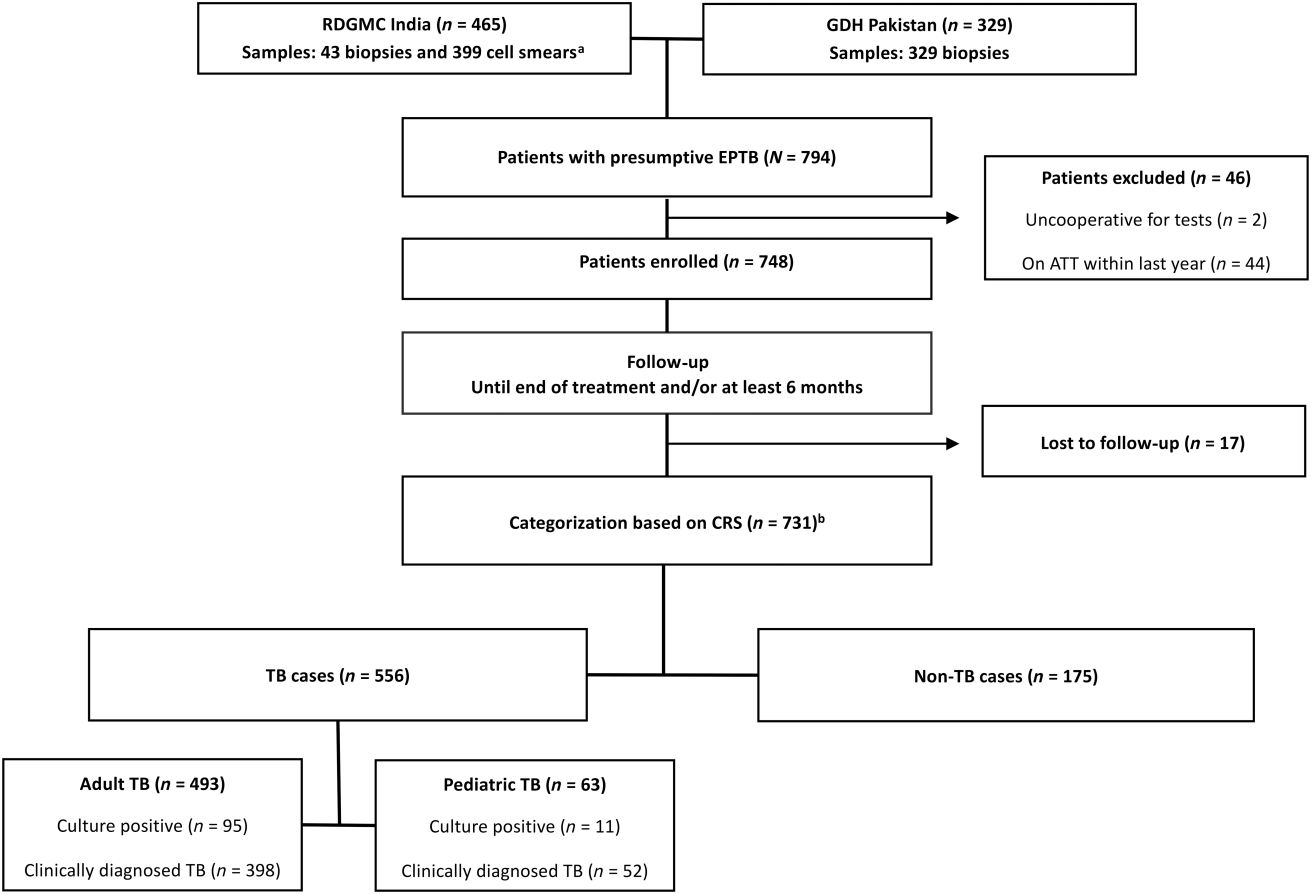
Flowchart of inclusion of patients and categorization. ^a^Thirteen cases with both biopsy and cytology samples. ^b^Categorization criteria: Culture positive; positive LJ culture or MGIT result. Clinically diagnosed; started on ATT and with a good response at 2/3 months or at end of treatment. Non‐TB; improvement without ATT or an alternative diagnosis made by the clinician. Non‐TB diagnoses are shown in supplementary material, Table S1. ATT, anti‐tuberculous treatment; CRS, composite reference standard; EPTB, extrapulmonary tuberculosis; GDH, Gulab Devi Hospital; LJ, Lowenstein–Jensen; MGIT; mycobacteria growth indicator tube; RDGMC, R.D. Gardi Medical College.

Samples were collected from patients at two tertiary care hospitals, Chandrikaben Rashmikant Gardi Hospital, associated with R.D. Gardi Medical College (RDGMC) in Ujjain, India, and Gulab Devi Hospital (GDH) in Lahore, Pakistan. New presumptive EPTB patients were prospectively enrolled from the inpatient or outpatient departments of the respective hospitals between April 2018 and February 2020 (Figure 1). Exclusion criteria were insufficient material for examination or receiving anti‐tuberculous treatment (ATT) at enrollment or within the past year. Seven biopsies were retrospectively included.

Demographic information was collected using a questionnaire. Patients were interviewed in Hindi, Urdu, or their local language. A clinical examination was performed, and blood samples were taken from all participants.

Patients were followed up regularly until completion of the treatment, or for at least 6 months. During follow‐up, clinical parameters were recorded to assess the response to treatment.

### Sample collection and processing

Samples were collected from the suspected sites of EPTB infection. Material for fine needle aspirates (FNA) from lymph nodes and superficial masses was collected using a 22G needle on a 10 ml syringe. Four smears were prepared, one for cytology, one for acid‐fast bacilli (AFB) detection, and two on flex slides for ICC. The slides for ICC were fixed in absolute ethanol for 1 h and stored at −20 °C until further processing. In addition, the material from the syringe was used for Xpert and Mtb culture. Fluids were aspirated aseptically and transported to the laboratory for Xpert and Mtb culture, and cell smears were prepared for AFB smear and ICC.

Fresh biopsies were cut in half, one half was fixed in formalin and sent for histopathology and IHC, whereas the other half was sent for AFB microscopy, Mtb culture, and Xpert. Most biopsies were already formalin‐fixed in blocks, from which 4‐μm‐thick sections were prepared for histopathology and IHC. At RDGMC, sputum was also collected from most of the patients for AFB microscopy, Xpert, and Mtb culture.

### Diagnostic tests

The Ziehl–Neelsen (ZN) staining method was used for the detection of AFB. Slides for cytologic and histopathologic examination were stained with May–Grünwald–Giemsa and hematoxylin–eosin stains, respectively. Cytomorphology compatible with TB was granulomatous inflammation with or without necrosis, or necrosis without predominance of neutrophils or caseous necrosis. In biopsies, granulomatous inflammation with or without necrosis, or caseous necrosis was considered consistent with TB.

Mycobacterial cultures were performed in the RDGMC or GDH microbiology laboratories. Samples were inoculated on Löwenstein–Jensen (LJ) medium and incubated at 37 °C for 8 weeks. At GDH, both LJ medium and mycobacteria growth indicator tube (MGIT) were used. Xpert was also performed at both sites according to WHO protocols [22].

### Immunostaining with MPT64

Pathologists (MRP, MK, and SI) were trained in immunostaining and slide evaluation. The production of the anti‐MPT64 antibodies used in our study is described in detail by Hoel *et al* [21]. In brief, rabbits were immunized to produce polyclonal anti‐MPT64 antibodies, and the performance of various adjuvants–antigen combinations was assessed by IHC screening using serial dilutions to identify the optimal working dilution. To overcome the limitation of batch‐to‐batch variability, the best performing individual sera with antibodies were pooled to produce one large volume of reproduced polyclonal antibodies. The antibodies are commercially available (anti‐MPT64, Cat# 157698, Ximbio, London, UK) [23].

In preparation for immunostaining, formalin‐fixed biopsies were deparaffinized with xylene before tissue sections and cell smears were rehydrated through decreasing concentrations of alcohol and washed with distilled water (see supplementary material, Protocols for immunochemical staining). Antigen retrieval was performed on biopsy specimens by heat‐induced antigen retrieval with citrate buffer, pH 6.2 using a microwave oven (300–400 W) for 15 min. To prevent endogenous peroxidase, tissue sections and cell smears were incubated with peroxidase block (hydrogen peroxide), 3% bovine serum albumin, and 10% normal goat serum. Slides were washed with buffer (Tris buffer, pH 7.6) between each step.

Immunostaining was performed as described previously [12, 16], but with some modifications (see supplementary material, Protocols for immunochemistry for full details). Slides were incubated for 1 h with polyclonal anti‐MPT64 primary antibodies at dilutions of 1/300 for IHC at RDGMC and 1/250 for IHC at GDH, and 1/200 for ICC together with the Dako Envision + System HRP kit (K4009, Dako, Glostrup, Denmark). In‐house methods were used to decide the optimal working dilutions at the different study sites depending on sample type and local conditions. A horseradish peroxidase‐conjugated secondary anti‐rabbit antibody was then applied for 45 min. The substrates 3‐amino‐9‐ethylcarbazol or diaminobenzidine were applied to the slides for 10 min for cell smears and for 15 min for biopsies. This was followed by counterstaining with hematoxylin and mounting with Immu‐Mount (Thermo Fisher Scientific, Waltham, MA, USA) or DPX (Merck KGaA, Darmstadt, Germany). The slides were washed with a wash buffer between incubation steps.

### Evaluation of immunostaining

**Figure 2:**
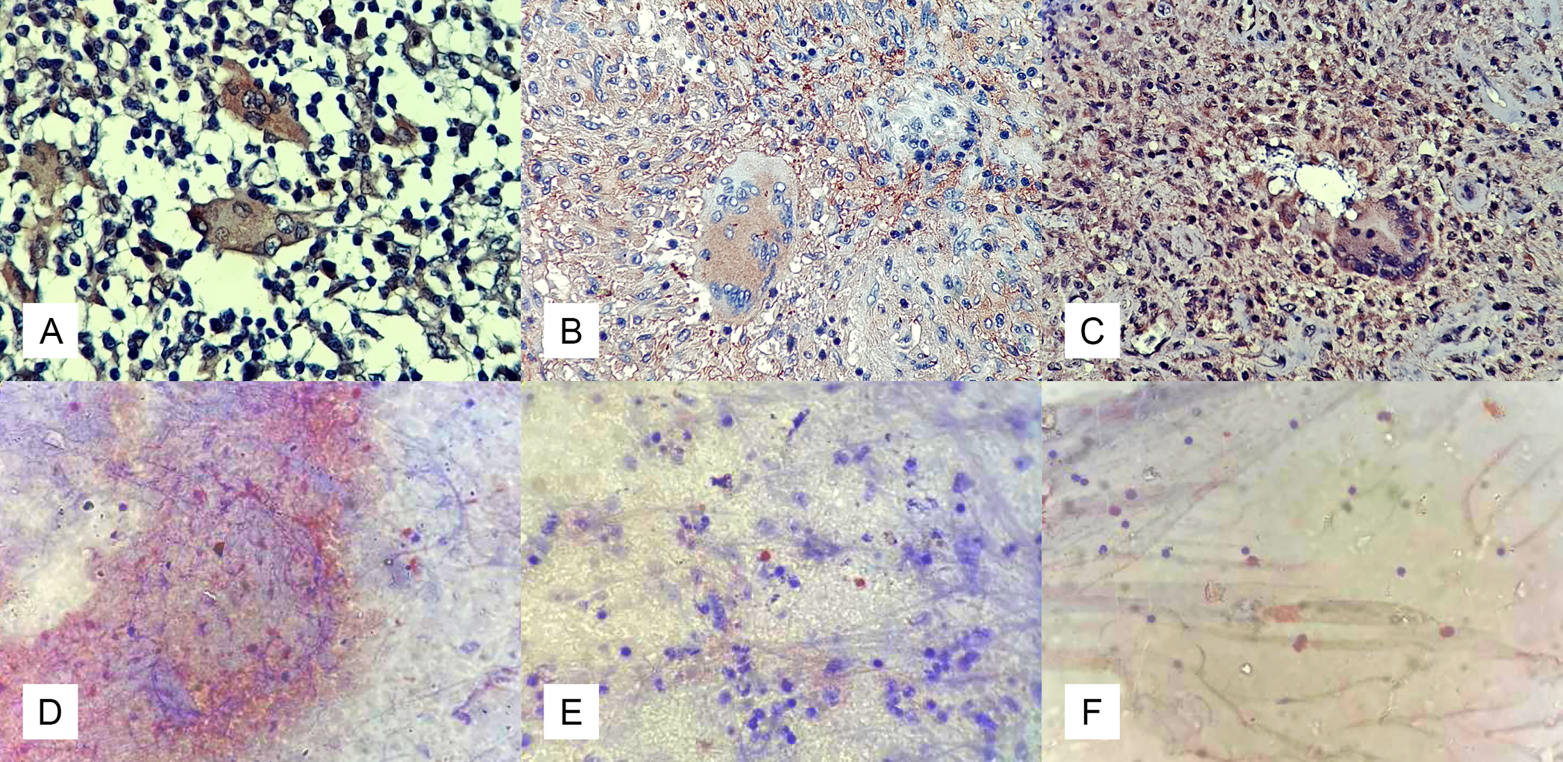
Positive MPT64 staining shown as brown‐reddish granular staining. (A) Positive immunohistochemical staining in Langhans type giant cells and lymphocytes from a biopsy of breast tissue. (B) Granuloma with positive immunohistochemical staining of giant cells, epithelioid cells, lymphocytes, and necrotic area in a biopsy from the omentum. (C) Positive immunohistochemical staining with strong signal in Langhans type giant cells, lymphocytes, and necrotic area in biopsy from peripheral lymph node. (D) Positive immunocytochemical staining in lymphocytes and necrotic background in fine needle aspirate from peripheral lymph node. (E) Positive immunocytochemical staining in necrotic area in fine needle aspirate from peripheral lymph node. (F) Positive immunocytochemical staining in lymphocytes in pleural fluid.

Immunostained slides were evaluated by RDGMC and GDH pathologists (MRP, MK, and SI), who were blinded for the categorization of patients. Slides were screened at ×10 magnification, and a more detailed assessment was performed at ×40 magnification. Slides were classified as positive if brown‐reddish granular staining was seen intracytoplasmically in inflammatory cells or extracellularly in necrotic areas (Figure 2).

### Categorization of patients

A CRS was used to categorize study participants into the culture confirmed TB, clinically diagnosed TB and non‐TB groups based on various diagnostic criteria (Figure 1). In brief, culture‐positive patients showed a positive result on LJ medium or MGIT. Clinically diagnosed cases had signs and symptoms suggestive of EPTB and started ATT with a good response at 2/3 months and/or at the end of treatment. Non‐TB cases either improved without ATT or the clinician made an alternative diagnosis (supplementary material, Table S1).

### Statistical analysis

Statistical analysis was performed using the Statistical Package for the Social Sciences (SPSS) for Windows, version 28. The chi square test was performed to assess differences in categorical variables. Sensitivity, specificity, positive predictive values, negative predictive values, and diagnostic accuracy were calculated using cross‐tabulations. A *p* value <0.05 was considered statistically significant. The STARD guidelines for the reporting of diagnostic accuracy studies were followed as closely as possible [24].

## Results

### Study population

A total of 748 patients were enrolled in the study, and based on the CRS, 556 cases were categorized as TB patients [493 adults and 63 pediatric cases (<15 years of age)], whereas 175 cases were categorized as non‐TB patients (139 adults and 36 pediatric cases) (Figure 1). In the TB group, 106 cases were culture confirmed, whereas 450 patients were clinically diagnosed. A significantly higher proportion of TB patients were female. Among adults, TB patients were younger compared with non‐TB patients (Table 1). The HIV prevalence was low in both groups.

**Table 1:** Demographic and baseline characteristics of 731 study participants

	Adults			Children		
	TB (*N* = 493)	Non‐TB (*N* = 139)	*p* value	TB (*N* = 63)	Non‐TB (*N* = 36)	*p* value
	*n* (%)	*n* (%)		*n* (%)	*n* (%)	
Age (years)						
<5				2 (3)	6 (17)	**<0.001**
5–9				16 (25.5)	18 (50)	
10–14				45 (71.5)	12 (33)	
15–29	293 (59)	46 (33)	**<0.001**			
30–44	128 (26)	35 (25)				
>45	72 (15)	58 (42)				
Gender						
Female	275 (56)	61 (44)	**0.013**	43 (68)	14 (39)	**0.004**
Male	218 (44)	78 (56)		20 (32)	22 (61)	
HIV positive*	11/457 (2)	4/137 (3)	0.757	0 (–)	0 (–)	–
Site of infection						
Lymphadenitis	340 (69)	80 (57.5)	**0.012**	56 (89)	27 (75)	0.071
Pleuritis	78 (16)	26 (18.5)	0.418	2 (3)	1 (3)	1.0
Meningitis	19 (4)	25 (18)	**<0.001**	1 (2)	7 (19)	**0.003**
Gastrointestinal	10 (2)	1 (1)	0.471	–	–	–
Other^†^	46 (9)	7 (5)	0.107	4 (6)	1 (3)	0.650
Sample type^‡^						
Biopsy	304 (62)	16 (12)	**<0.001**	41 (65)	1 (3)	**<0.001**
Cytology	202 (41)	123 (88)		22 (35)	35 (97)	
Diagnostic tests						
Xpert positive^§^	96/250 (38)	0/125 (0)	**<0.001**	11/32 (34)	0/35 (0)	**<0.001**
Culture positive^‖^	95/284 (33)	0/123 (0)	**<0.001**	11/34 (32)	0/33 (0)	**<0.001**
ZN positive^¶^	59/446 (13)	1/130 (1)	**<0.001**	7/58 (12)	0/35 (0)	**0.033**

TB and non‐TB cases as per CRS. Significant *p* values are shown in bold.

HIV, human immunodeficiency virus; Xpert, GeneXpert MTB/RIF; ZN, Ziehl–Neelsen.

* Results available from 680 samples.

^†^ TB adults: abscess (*n* = 24), urogenital (*n* = 8), osteomyelitis (*n* = 6), breast (*n* = 3), skin (*n* = 3), pericarditis (*n* = 1), synovial fluid (*n* = 1). TB children: abscess (*n* = 2), urogenital (*n* = 1), laryngitis (*n* = 1). Non‐TB adults: abscess (*n* = 3), urogenital (*n* = 2), osteomyelitis (*n* = 1), breast (*n* = 1). Non‐TB children: osteomyelitis (*n* = 1).

^‡^ Among CRS positive adults, 13 samples have both cytology and biopsy results, thus total percentage exceeds 100% for this group.

^§^ Valid results available from 442 samples.

^‖^ Valid results available from 474 samples.

^¶^ Valid results available from 669 samples.

Adenitis was the most common form of infection in both groups, accounting for 71% and 61% of TB and non‐TB cases, respectively (Table 1). Most patients in the non‐TB group improved without ATT and without a reported alternative diagnosis. The most common non‐TB diagnoses were abscess (*n* = 20), benign tumors (*n* = 10), and malignancies (*n* = 8) (supplementary material, Table S1).

### Performance of all diagnostic tests on biopsies

In biopsies, the MPT64 test showed good diagnostic performance, with an overall sensitivity of 94% (95% CI: 91–97) against a CRS (Tables 2 and 3). The sensitivity was 49% (95% CI: 39–59) for Mtb culture and 69% (95% CI: 56–80) for Xpert. Most biopsies were from lymph nodes and pleural tissue, where the MPT64 test demonstrated good true positive rates of 96% and 94%, respectively (Table 2). The overall specificity of the test in biopsies was 75% (95% CI: 43–95). Positive predictive values, negative predictive values, and diagnostic accuracy were calculated based on the cohort prevalence and are presented in supplementary material, Table S2. For biopsy specimens, the correlation between a positive MPT64 test result and TB histology was good at 95% (supplementary material, Table S3).

**Table 2:** Results of diagnostic tests in pediatric and adult extrapulmonary samples

	ZN	Culture	Xpert	MPT64
**TB samples (*N* = 556)**	66/503 (13)	106/318 (33)	107/282 (38)	392/539 (73)
All biopsy samples (*N* = 345)	33/293 (11)	52/106 (49)	42/61 (69)	320/339 (94)
Adult biopsies	27/257 (11)	45/93 (48)	36/51 (71)	282/299 (94)
Pediatric biopsies	6/36 (17)	7/13 (54)	6/10 (60)	38/40 (95)
Sites, all ages				
Lymph node biopsies	29/232 (13)	50/91 (55)	25/39 (64)	244/255 (96)
Pleural biopsies	3/28 (11)	0/7 (0)	6/9 (67)	31/33 (94)
Other sites*, biopsies	1/33 (3)	2/8 (25)	11/13 (85)	45/51 (88)
All cytology samples (*N* = 224)	33/224 (15)	55/212 (26)	65/221 (29)	76/212 (36)
Adult cytology samples	32/202 (16)	51/191 (27)	60/199 (30)	71/191 (37)
Pediatric cytology samples	1/22 (5)	4/21 (19)	5/22 (23)	5/21 (24)
Sites, all ages				
FNA lymph nodes	28/148 (19)	42/137 (31)	50/149 (34)	61/142 (43)
Pleural effusions	4/46 (9)	7/45 (16)	8/44 (18)	13/45 (29)
CSF	0/20 (0)	2/20 (10)	3/20 (15)	3/15 (20)
Other sites^†^, cytology	1/10 (10)	4/10 (40)	4/9 (44)	3/10 (30)
**Non‐TB samples (*N* = 175)**	1/165 (0.6)	0/156 (0)	0/160 (0)	31/156 (20)
All biopsy samples (*N* = 17)	0/7 (0)	0/2 (0)	0/3 (0)	3/12 (25)
Adult biopsies	0/7 (0)	0/2 (0)	0/3 (0)	2/11 (18)
Pediatric biopsies	–	–	–	1/1 (100)
Sites, all ages				
Lymph node biopsies	0/1 (0)	–	–	0/1 (0)
Pleural biopsies	0/4 (0)	0/1 (0)	0/2 (0)	1/4 (25)
Other sites^‡^, biopsies	0/2 (0)	0/1 (0)	0/1 (0)	2/7 (29)
All cytology samples (*N* = 158)	1/158 (0.6)	0/154 (0)	0/157 (0)	28/144 (19)
Adult cytology samples	1/123 (0.8)	0/121 (0)	0/122 (0)	25/112 (22)
Pediatric cytology samples	0/35 (0)	0/33 (0)	0/35 (0)	3/32 (9)
Sites, all ages				
FNA lymph nodes	1/106 (0.9)	0/102 (0)	0 (0)	20/101 (20)
Pleural effusions	0/19 (0)	0/19 (0)	0/18 (0)	6/19 (32)
CSF	0/32 (0)	0/32 (0)	0/32 (0)	2/23 (9)
Other sites^§^, cytology	–	–	–	0/1 (0)

TB and non‐TB cases as per CRS. Data are *n*/*N* (%). Thirteen TB cases had MPT64 results from both biopsy and cytology samples.

CSF, cerebrospinal fluid; Culture, *Mycobacterium tuberculosis* culture; FNA, fine needle aspirate; Xpert, GeneXpert MTB/RIF; ZN, Ziehl–Neelsen.

* Abscess (*n* = 24), urogenital (*n* = 9), bones (*n* = 6), gastrointestinal (*n* = 6), skin (*n* = 3), pericardial (*n* = 1), larynx (*n* = 1), breast (*n* = 1).

^†^ Ascites (*n* = 5), pus (*n* = 3), abscess (*n* = 1), synovial fluid (*n* = 1).

^‡^ Abscess (*n* = 3), bones (*n* = 2), urogenital (*n* = 2), breast (*n* = 1).

^§^ Ascitic fluid (*n* = 1).

**Table 3:** Diagnostic validation of tests for EPTB, compared to a CRS and culture

	Against CRS			Against culture		
	*n*	Sensitivity (95% CI)	Specificity (95% CI)	*n*	Sensitivity (95% CI)	Specificity (95% CI)
**Biopsy samples**						
All patients						
Xpert	64	69 (56–80)	100 (29–100)	35	71 (29–96)	29 (13–49)
Culture	108	49 (39–59)	100 (16–100)	–	–	–
ZN microscopy	300	11 (8–15)	100 (53–100)	94	20 (10–35)	90 (78–97)
MPT64	351	94 (91–97)	75 (43–95)	107	100 (93–100)	2 (0.05–10)
Xpert and/or MPT64	353	94 (91–97)	75 (43–95)	107	100 (93–100)	2 (0.05–10)
Adults						
Xpert	54	71 (56–83)	100 (29–100)	29	80 (28–99)	29 (13–51)
Culture	95	48 (38–59)	100 (16–100)	–	–	–
ZN microscopy	264	11 (7–15)	100 (59–100)	83	13 (4–27)	88 (74–96)
MPT64	310	94 (91–97)	82 (48–98)	94	100 (92–100)	–^†^
Xpert and/or MPT64	312	94 (91–97)	82 (48–98)	94	100 (92–100)	–^†^
Pediatric cases						
Xpert	10	60 (26–88)	–*	6	50 (1–99)	25 (1–81)
Culture	13	54 (25–81)	–*	–	–	–
ZN microscopy	36	17 (6–33)	–*	11	80 (28–99)	100 (54–100)
MPT64	41	95 (83–99)	0 (0–97)	13	100 (59–100)	17 (0.4–64)
Xpert and/or MPT64	41	95 (83–99)	0 (0–98)	13	100 (59–100)	17 (0.4–64)
**Cytology samples**						
All patients						
Xpert	378	29 (23–36)	100 (98–100)	362	93 (82–98)	96 (93–98)
Culture	366	26 (20–32)	100 (98–100)	–	–	–
ZN microscopy	382	15 (10–20)	99 (97–100)	366	47 (32–59)	98 (96–99)
MPT64	356	36 (29–43)	81 (73–87)	340	46 (33–60)	75 (70–80)
Xpert and/or MPT64	382	50 (44–57)	82 (75–88)	366	95 (85–98)	74 (69–79)
Adults						
Xpert	321	30 (24–37)	100 (97–100)	308	92 (81–98)	95 (92–98)
Culture	312	27 (21–34)	100 (97–100)	–	–	–
ZN microscopy	325	16 (11–22)	99 (96–100)	312	49 (35–63)	98 (96–99)
MPT64	303	37 (30–44)	78 (69–85)	290	46 (32–61)	73 (67–78)
Xpert and/or MPT64	325	52 (45–59)	80 (71–86)	312	94 (84–99)	72 (66–77)
Pediatric cases						
Xpert	57	23 (8–45)	100 (90–100)	54	100 (40–100)	98 (89–100)
Culture	54	19 (5–42)	100 (89–100)	–	–	–
ZN microscopy	57	5 (0.1–23)	100 (90–100)	54	25 (0.6–81)	100 (93–100)
MPT64	53	24 (8–47)	91 (75–98)	50	50 (7–93)	87 (74–95)
Xpert and/or MPT64	57	36 (17–59)	91 (77–98)	54	100 (40–100)	86 (73–94)

CI, confidence interval; CRS, composite reference standard; EPTB, extrapulmonary tuberculosis; Xpert, GeneXpert MTB/RIF; ZN, Ziehl‐Neelsen.

* No non‐TB cases in pediatric biopsy cases for Xpert, culture, and ZN when using a CRS.

^†^ No non‐TB cases in this group.

### Comparison of adult and pediatric biopsy cases

In biopsies, the performance of the MPT64 test was similar between adults and children, with a sensitivity of 94% (95% CI: 91–97) for adults and 95% (95% CI: 83–99) for children (Table 3). Likewise, the performance of ZN, Xpert, and Mtb culture did not differ significantly between adults and children.

### Performance of all diagnostic tests on cytology samples

Against a CRS, the sensitivity and specificity of the MPT64 test for cytology specimens were 36% (95% CI: 29–43) and 81% (95% CI: 73–87), respectively (Tables 2 and 3). The test performed best in FNA samples from lymph nodes (sensitivity of 43%), whereas the true positive rate was lower in samples from pleural fluid and cerebrospinal fluid (CSF) (Table 2). Specificity was good in CSF samples at 91% and in FNA at 80% (Table 2). Overall, the sensitivity of the MPT64 test in cytology samples was better than the routine diagnostic tests ZN microscopy, Mtb culture, and Xpert, which had true positive rates of 15% (95% CI: 10–20), 26% (95% CI: 20–32), and 29% (95% CI: 23–36), respectively (Table 3). When a positive Xpert result and/or a positive MPT64 result was added, the sensitivity increased to 50% (95% CI: 44–57), while the specificity was 82% (95% CI: 75–88) when using CRS as the reference standard (Table 3). The added diagnostic yield of the MPT64 test and Xpert combined in cytology samples was 95% (95% CI: 85–98) in culture positive samples.

Surprisingly, the correlation between cytomorphology suggestive of tuberculous lymphadenitis (TBLA) and a positive MPT64 result was low (45%) (supplementary material, Table S3).

### Comparison of adult and pediatric cytology cases

In cytology specimens, the MPT64 test showed a nonsignificantly higher sensitivity in adults compared with pediatric cases [37% (95% CI: 30–44) versus 24% (95% CI: 8–47)] (Table 3) while the specificity was higher among pediatric samples [78% (95% CI: 69–86) versus 91% (95% CI: 75–98)]. The performance of ZN, Xpert, and Mtb culture was similar in the two groups.

### Comparison of diagnostic tests

**Figure 3:**
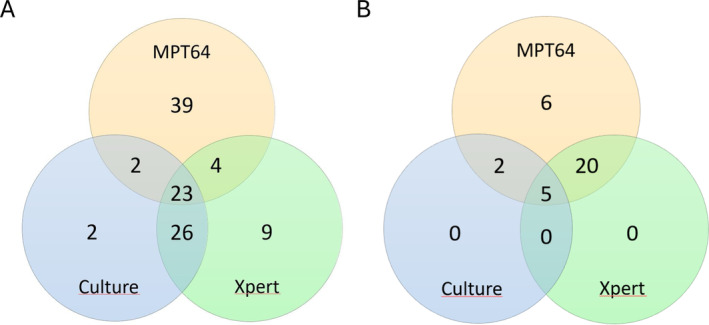
Head‐to‐head comparison of diagnostic tests for extrapulmonary tuberculosis. Venn diagrams including samples with valid results from all three tests. (A) Cytology samples (*n* = 105). Ziehl–Neelsen (ZN) microscopy was positive in 30 samples. (B) Biopsy samples (*n* = 33). ZN microscopy was positive in three samples.

The Venn diagram (Figure 3) shows a direct comparison of the diagnostic tests for EPTB in samples with valid results from all three tests. In cytology samples, the tests identified different patients, with 39 MPT64 positive cases being negative on Xpert and Mtb culture, while 23 patients were positive on all three tests. In biopsies with valid results from all tests, all Xpert and/or culture positive samples were also positive with the MPT64 test, with an additional six cases diagnosed with the MPT64 test only.

## Discussion

This is the first study to evaluate the diagnostic accuracy of the MPT64 antigen detection test on cytology and biopsy specimens by ICC and IHC using a reproduced batch of polyclonal antibodies. Against a CRS, the overall sensitivity of the test was better than Xpert and culture. The test performed best on biopsy specimens with a sensitivity of 94%, and results were equally good in adults and children. In cell smears from pus, FNA, and effusions, the sensitivity of the MPT64 test was low but higher than Xpert and culture against a CRS. The specificity of the MPT64 test was particularly good in pediatric cytology specimens (91%), and lower in adults (78%). Thus, the MPT64 test in combination with routine diagnostics can increase the diagnostic yield for EPTB in high burden settings.

Although primarily considered a pulmonary disease, about 17% of the estimated 10.6 million annual global TB cases are extrapulmonary [25]. However, this may be a low estimate because of the difficulty in confirming EPTB diagnosis and the reporting of coexisting extrapulmonary and pulmonary TB disease as pulmonary TB only in many high burden countries. Furthermore, the proportion of EPTB is increasing in many regions [26, 27]. In Western Europe, for example, up to 53% of TB cases are extrapulmonary [27]. Even though EPTB is not contagious, it still causes significant morbidity and has serious economic consequences for patients and their families [28, 29]. Improving EPTB diagnosis is therefore important in both low‐ and high‐prevalence areas.

The sensitivity of the MPT64 test in biopsies was good (94%), confirming previous studies [12, 13]. The MPT64 test performed better than AFB microscopy, Xpert, and Mtb culture with sensitivities of 11%, 49%, and 69%, respectively, against a CRS. We hypothesize that the superior sensitivity of MPT64 compared with Xpert and Mtb culture in our study could be explained by antigen accumulation in the host cells at the site of infection. This phenomenon has been described as a central process in TB pathogenesis and tissue destruction [30]. MPT64 antigen has been found to accumulate in granuloma structures, suggesting a role in the persistence of chronic infection [31]. Thus, the accumulated antigen in the lesions is the most plausible explanation for the superior performance of the MPT64 antigen detection test.

The advantage of the MPT64 test on biopsy specimens is not only in differentiating TB from other granulomatous diseases, but also in the detection of other diagnoses such as cancers by the histopathologic evaluation of the lesions. In a high TB burden setting, the main differential diagnoses for conditions with granulomatous inflammation are fungal infections, NTM infections, and sarcoidosis, with very different treatment options [6, 32]. Especially, NTM infections have become relevant in recent years due to the high prevalence in HIV‐infected individuals. As the MPT64 antigen is specific to the Mtb complex and is not present in NTM infections, the test could be useful to distinguish these diseases. Furthermore, our study showed a high correlation between histopathologic patterns suggestive of TB and a positive MPT64 result, suggesting that the MPT64 test in conjunction with histopathologic descriptions could aid the pathologist in diagnosing EPTB.

The MPT64 test may be useful in diagnosing ‘incidental’ TB, which commonly occurs in high prevalence settings due to the nonspecific clinical presentation of the various forms of EPTB [33, 34]. Postoperative biopsies are often fixed in formalin and therefore not suitable for Xpert investigation [5] and culture but can be evaluated by the MPT64 test. Furthermore, with logistic support, sampling could be done in peripheral areas and, after fixation, the slides could be transported to a central laboratory for evaluation. These unique features make the MPT64 test a useful supplement to routine diagnostics for EPTB.

The sensitivity of the MPT64 test in cytology specimens was low in our study (36%), contrasting the results of our previous studies, which showed sensitivities of 69–92% [18, 19, 20]. This could be explained by the difference in primary polyclonal antibodies. In all previous studies, we used an in‐house polyclonal antibody which was available in limited amounts. With the intention for scale‐up of the test, we reproduced the primary antibody and produced a single batch of this new polyclonal antibody in large amounts [21]. Since polyclonal antibodies recognize and bind to different epitopes of the antigen in question, the performance of the reproduced primary antibodies may differ from those previously used. In addition, the performance of the MPT64 test may be affected by differences in the *in vivo* epitope expression due to differences in fixation techniques and sample preparation. Formalin fixation is known to alter the three‐dimensional conformation the epitopes in biopsies [35] and, although antigen retrieval steps reverse this, it can still affect the epitope expression. For cytology specimens, alcohol fixation and the long storage time in a freezer could affect the epitope affinity. Although the overall performance of the MPT64 test on cytology samples was low, it still performed better than the routine diagnostic tests AFB microscopy, culture, and Xpert.

Fine needle aspiration cytology (FNAC) has become an important tool in diagnosing TB, especially in TBLA. However, the traditional definition of cytomorphology suggestive of TB is challenged by studies finding a high proportion of TB patients without the typical finding of granulomatous inflammation on FNAC [36]. Particularly, FNAC samples showing necrosis without granulomas are a major challenge for the pathologist to rule out TB in an endemic setting. The MPT64 test correctly identified a large proportion (76%) of TB patients with necrosis without granulomatous inflammation on cytology (supplementary material, Table S3) and combining the MPT64 result with cytologic descriptions increased the diagnostic yield (supplementary material, Table S4). Surprisingly, the specificity of the test was low in FNA samples with cytology suggestive of TB, adding little value in confirming TB in this patient group (supplementary material, Table S3). In a TB endemic setting, some false positive results could be due to concomitant TB antigens in the samples of non‐TB cases. Furthermore, the natural course of TBLA may be relapsing and remitting [37], which could lead to misclassification of non‐TB patients. However, despite its limitations, FNAC is an important tool in the diagnosis of EPTB in a high burden setting due to its simplicity and good sensitivity.

The performance of the MPT64 test was similar between adult and pediatric cases in our study. Childhood TB has been largely neglected in the literature and poses a particular diagnostic challenge [38, 39]. The potential role of the MPT64 test as a confirmatory test for pediatric TBLA has been suggested based on promising results [19, 20]. In our study, the sensitivity of the MPT64 test for diagnosing TBLA in children was only 25% (supplementary material, Table S4), but the specificity was excellent at 96%, suggesting an additive value of the MPT64 test in diagnosing pediatric TBLA.

The MPT64 test performed better than Xpert in our study, compared with a CRS. WHO‐endorsed rapid molecular tests such as the Xpert/Xpert Ultra and TrueNat (Molbio, Goa, India) are recommended for the timely diagnosis of TB [40]. However, these tests do not perform equally well on all sample types, limiting the use for most forms of EPTB [4]. The MPT64 test, although not as rapid as Xpert, could provide a result within 1–2 days, which could significantly reduce the diagnostic delay in EPTB. We found that adding the results of Xpert and the MPT64 test increased the diagnostic yield for all types of EPTB. Our findings support the use of the MPT64 test along with molecular testing to increase sensitivity in presumptive EPTB while still providing a rapid diagnosis.

The MPT64 test, Xpert, and culture identified different patients and thus complemented each other in cytology samples. Early bacterial multiplication before sufficient antigen accumulation could explain some of the cases with a positive culture result but negative immunostaining. By combining molecular tests detecting DNA with antigen detection tests, the specimens are subjected to two fundamentally different diagnostic methods, which could increase the diagnostic yield for EPTB.

We found good correlation of test performance on biopsies between the two study sites, suggesting that the MPT64 test is robust and reliable between sites (supplementary material, Table S5). The test depends on invasive sampling and basic laboratory facilities being available, which are not always present in rural health care facilities in endemic countries. However, prioritizing the strengthening of laboratory structures would improve the health care services in general, and increase the diagnostic possibilities for a wide range of diseases. Furthermore, the immunochemical staining technique is a valuable asset in the diagnosis of other conditions, most importantly cancers.

The major strengths of this study are the high number of EPTB cases enrolled at two centers with good laboratory facilities in high TB burden settings, the long follow‐up period, and inclusion of clinically diagnosed cases. However, there are limitations to our study. The use of a CRS to compensate for the imperfection of culture in diagnosing EPTB comes with the risk of misclassification. Especially the use of the rather broad antibiotic rifampicin in first‐line ATT could lead to patients with other bacterial infection being misclassified as TB patients. In addition, many cases labeled as non‐TB lacked an alternative diagnosis, which could particularly affect the specificity of the test. Furthermore, not all samples were subjected to the same number of reference tests, which could overestimate and underestimate the diagnostic performance of the test. In addition, the COVID‐19 pandemic temporarily disrupted the supply of key reagents for MPT64 immunochemistry, resulting in prolonged storage of samples in a freezer, which could influence epitope presentation.

In conclusion, diagnosing EPTB is challenging and there is a need for improved diagnostics, especially for childhood EPTB. We have shown that the MPT64 antigen detection test has a higher diagnostic yield than Xpert and culture in diagnosing EPTB in high burden settings against a CRS. The test performed particularly well on biopsy specimens and may aid pathologists to diagnose EPTB in conjunction with routine TB diagnostics and histopathology. Although sensitivity was low in cytology samples, the high specificity in pediatric FNA specimens suggests a role for the MPT64 test to support the diagnosis of childhood TB. We found an additive value of combining the MPT64 test and Xpert in diagnosing EPTB. Further feasibility studies and cost–benefit analyses are needed to determine the potential role of the MPT64 test in the routine diagnosis of EPTB.

## Author contributions statement

OMBH performed data curation, formal analysis, wrote the first draft and finalized the manuscript. MK performed data collection, investigations, sampling, interpretation of the staining and reviewed the manuscript. SI performed data collection, description and interpretation of the staining and reviewed the manuscript. AA assisted with data collection, microbiological analysis and edited and reviewed the manuscript. MRP organized the study, contributed toward data collection, sampling, description and interpretation of the staining and edited the manuscript. TM conceptualized the study, acquired funds for the study, developed the methodology, developed the analysis plan, supervised and edited the manuscript. All authors were involved in writing the paper and had final approval of the submitted and published versions.

## Supporting information


**Table S1.** CRS and categorization of patients
**Table S2.** Diagnostic validation of tests for EPTB in adults and children
**Table S3.** Positive MPT64 results and their correlation to cytomorphologic and histopathologic descriptions
**Table S4.** Diagnostic accuracy of cytology, routine TB diagnostics, and the MPT64 test on FNA samples in adults and children
**Table S5.** Comparison of MPT64 diagnostic accuracy in biopsies from Pakistan and India
Protocols for immunochemical staining at RDGMC and GDH

## Data Availability

Data generated and analyzed during the study are available in the article and supplementary material. Further details are available from the corresponding author upon reasonable request.
