# Supplementary material for: Diagnosing adult and pediatric extrapulmonary tuberculosis by MPT64 antigen detection with immunohistochemistry and immunocytochemistry using reproduced polyclonal antibodies
Source: J Pathol Clin Res. 2024 Apr 4;10(3):e12373. doi: 10.1002/2056-4538.12373 (PMC10993049; doi:10.1002/2056-4538.12373)
Supplement: Supplementary file 1 — Table S1. CRS and categorization of patients Table S2. Diagnostic validation of tests for EPTB in adults and children Table S3. Positive MPT64 results and their correlation to cytomorphologic and histopathologic descriptions Table S4. Diagnostic accuracy of cytology, routine TB diagnostics, and the MPT64 test on FNA samples in adults and children Table S5. Comparison of MPT64 diagnostic accuracy in biopsies from Pakistan and India Protocols for immunochemical staining at RDGMC and GDH [file CJP2-10-e12373-s001.pdf]

Diagnosing adult and pediatric extrapulmonary tuberculosis by MPT64 antigen detection with immunohistochemistry and immunocytochemistry using reproduced polyclonal antibodies

OMB Helle *et al*., *J Pathol Clin* Res, https://doi.org/10.1002/2056-4538.12373

Supplementary Tables S1–S5

Protocols for immunochemical staining at RDGMC and GDH

Table S1. Composite reference standard and categorization of patients	
**Culture confirmed TB case (n = 106)**	Positive *mycobacterium tuberculosis* culture
**Probable TB case (n = 243)**	Clinical presumptive EPTB patient started on anti-tuberculous treatment (ATT) with a good response[a] at 2/3 months and/or at end of treatment, **and at least one of the following additional criteria:**<br>a) Positive Xpert MTB/Rif assay<br>b) Extrapulmonary material positive for AFB smear<br>c) Radiological findings suggestive of EPTB[b]<br>d) Concomitant pulmonary TB suggested by positive AFB smear or bacteriologically confirmed<br>e) Effusions/CSF: lymphocytosis on fluid cytology and protein level > 3 g/dl (> 1 g/l for CSF) or Adenosine deaminase (ADA) ≥ 40 IU/L (≥10 IU/L for CSF)<br>f) FNAC/Biopsy with morphological features suggestive of TB[c]
**Possible TB case (n = 207)**	Clinical presumptive EPTB patient started on anti-tuberculous treatment (ATT) with a good response[a] at 2/3 months and/or at end of treatment[d]
**Non-TB case (n = 175)[e]**	Patient started on ATT based on clinical presumptive EPTB but did not respond to treatment<br>OR<br>Improvement without ATT and/or response to specific non-tuberculous therapy<br>OR<br>Alternative diagnosis concluded by the clinician
**Uncategorized patient**	Not possible to categorize/loss to follow-up before 1st follow-up.

[a]A good response to treatment in cytology cases was considered when a minimum of three of the following was recorded at 2/3 months and/or at end of treatment:

- improvement in systemic and local symptoms,
- weight gain
- reduction of lymph node size or improvement of local finding (i.e. reduction of pleural fluid on CXR)
- improvement in subjective score using VAS and EQ-5D

[b] Pleural effusion or findings suggesting miliary TB on chest X-ray

[c] Finding of granulomatous inflammation with or without necrosis or only necrosis (caseous or liquefactive)

[d] Thirty-one biopsy cases from Pakistan did not have data on response to treatment but showed morphology suggestive of TB.

[e] Non - TB diagnoses:
Improved without anti-tuberculous treatment (no other diagnosis specified) (n = 93), Abscess (n = 20), Malignancies (n = 8), Benign tumor (n = 10), Fungal infection (n = 8), Other bacteriological infection (including NTM) (n = 7), CVA or cardiac arrest (n = 6), Chronic inflammation (n = 4), Other neurological conditions (n = 4), Liver cirrhosis with encephalopathy (n = 3), Unknown (n = 6), Other (n = 6, mastitis, prostate hyperplasia, intoxication, hyperglycemia, COPD, lumbar degenerative disc disease)

Table S2. Diagnostic validation of tests for EPTB							
	**n**	**Sensitivity (95% CI)**	**Specificity (95% CI)**	**PPV (95% CI)**	**NPV (95% CI)**	**Accuracy (95% CI)**	
**Cytology samples**							
**All patients, against CRS [a]**							
Xpert	378	29 (23 – 36)	100 (98 – 100)	100 (94 – 100)	51 (49 – 53)	59 (54 – 64)	TP 65, FP 0 FN 156, TN 157
Culture	366	26 (20 – 32)	100 (98 -100)	100 (94 – 100)	49 (47 – 51)	57 (52 – 62)	TP 55, FP 0 FN 157, TN 154
ZN microscopy	382	15 (10 – 20)	99 (97 – 100)	97 (82 – 100)	46 (44 – 47)	50 (45 – 55)	TP 33, FP 1 FN 191, TN 157
MPT64	356	36 (29 – 43)	81 (73 – 87)	72 (64 - 79)	48 (44 - 51)	55 (49 - 60)	TP 76, FP 28 FN 136, TN 116
Xpert and/or MPT64	382	50 (44 – 57)	82 (75 – 88)	80 (73 – 85)	55 (51 – 58)	64 (59 – 69)	TP 113, FP 28 FN 111, TN 130
**All patients, against culture**							
Xpert	362	93 (82 – 98)	96 (93 – 98)	97 (95 – 98)	90 (78 – 96)	94 (91 – 96)	TP 50, FP 13 FN 4, TN 295
ZN microscopy	366	47 (34 – 61)	98 (96 – 99)	98 (94 – 99)	57 (51 – 63)	69 (64 – 73)	TP 26, FP 5 FN 29, TN 306
MPT64	340	46 (33 - 60)	75 (70 – 80)	72 (64 - 79)	50 (44 – 57)	58 (53 – 64)	TP 25, FP 71 FN 29, TN 215
Xpert and/or MPT64	366	95 (85 – 99)	74 (69 – 79)	84 (81 - 86)	91 (77 – 97)	86 (82 – 89)	TP 52, FP 80 FN 3, TN 231
**Pediatric cases, CRS [b]**							
Xpert	57	23 (8-45)	100 (90-100)	100 (48 – 100)	67 (62 – 72)	70 (56 – 81)	TP 5, FP 0 FN 17, TN 35
Culture	54	19 (5-42)	100 (89 - 100)	100 (40 – 100)	66 (61 – 70)	68 (54 – 80)	TP 4, FP 0 FN 17, TN 33
ZN microscopy	57	5 (0.1-23)	100 (90-100)	100 (3 – 100)	62 (60 – 64)	63 (49 – 75)	TP 1, FP 0 FN 21, TN 35
MPT64	53	24 (8 -47)	91 (75 - 98)	62 (30 – 86)	65 (59 – 71)	65 (50 – 77)	TP 5, FP 3 FN 16, TN 29
Xpert and/or MPT64	57	36 (17 - 59)	91 (77 – 98)	73 (45 – 90)	69 (62 – 76)	70 (56 – 81)	TP 8, FP 3 FN 14, TN 32
**Pediatric cases, against culture**							
Xpert	54	100 (40-100)	98 (89-100)	97 (82 – 100)	100 (93 -100)	99 (91 – 100)	TP 4, FP 1 FN 0, TN 49
ZN microscopy	54	25 (.6- 81)	100 (93 – 100)	100 (3 – 100)	68 (54 – 79)	71 (57 – 82)	TP 1, FP 0 FN 3, TN 50
MPT64	50	50 (7- 93)	87 (74 – 95)	71 (42 – 89)	73 (50 – 88)	73 (58 – 84)	TP 2, FP 6 FN 2, TN 40
Xpert and/or MPT64	54	100 (40 – 100)	86 (73 – 94)	82 (70 – 90)	100 (92 – 100)	91 (81 – 97)	TP 4, FP 7 FN 0, TN 43
**Adults, against CRS[c]**							
Xpert	321	30 (24 – 37)	100 (97 – 100)	100 (94 – 100)	47 (45 – 49)	57 (51 – 62)	TP 60, FP 0 FN 139, 122
Culture	312	27 (21 – 34)	100 (97 – 100)	100 (93 – 100)	43 (41 – 46)	53 (47 – 59)	TP 51, FP 0 FN 140, TN 121
ZN microscopy	325	16 (11 – 22)	99 (96 – 100)	97 (81 – 100)	42 (40 – 43)	48 (42 – 53)	TP 32, FP 1 FN 170, TN 122
MPT64	303	37 (30 – 44)	78 (69 – 85)	73 (65- 80)	43 (40 – 47)	53 (47 – 58)	TP 71, FP 25 FN 120, TN 87
Xpert and/or MPT64	325	52 (45 – 59)	80 (71 – 86)	81 (74 – 86)	50 (46 – 55)	63 (57 – 68)	TP 105, FP 25 FN 97, TN 98
**Adults, against culture**							
Xpert	308	92 (81 – 98)	95 (92 – 98)	97 (95 – 98)	87 (72 – 95)	93 (90 – 96)	
ZN microscopy	312	49 (35 – 63)	98 (96 – 99)	98 (95– 99)	52 (45 – 59)	67 (61 – 72)	TP 25, FP 5 FN 26, TN 256

MPT64	290	46 (32 – 61)	73 (67 – 78)	75 (68 – 81)	43 (37 – 50)	56 (50 – 62)	TP 23, FP 65<br>FN 27, TN 175
Xpert and/or MPT64	312	94 (84 – 99)	72 (66 – 77)	86 (83 – 88)	87 (70 – 95)	86 (82 – 90)	TP 48, FP 73<br>FN 3, TN 188
**Biopsies**							
**All patients, against CRS[d]**							
Xpert	64	69 (56 – 80)	100 (29 – 100)	100 (92 – 100)	14 (10 – 20)	70 (58 - 81)	TP 42, FP 0<br>FN 19, TN 3
Culture	108	49 (39 – 59)	100 (16 - 100)	100 (93 – 100)	9 (8 - 11)	52 (42 – 61)	TP 52, FP 0<br>FN 54, TN 2
ZN microscopy	300	11 (8 – 15)	100 (53 – 100)	100 (89 – 100)	6 (5 – 6)	16 (12 – 20)	TP 33, FP 0<br>FN 260, TN 7
MPT64	351	94 (91 - 97)	75 (43 - 95)	99 (96 – 100)	41 (29 -55)	93 (90 – 96)	TP 320, FP 3<br>FN 19, TN 9
Xpert and/or MPT64	353	94 (91 – 97)	75 (43 – 95)	99 (96 – 99)	41 (29 – 55)	93 (90 – 96)	TP 322, FP 3<br>FN 19, TN 9
**All patients, against culture**							
Xpert	35	71 (29 – 96)	29 (13 – 49)	95 (92 - 97)	5 (1 – 16)	69 (51 – 84)	TP 5, FP 20<br>FN 2, TN 8
ZN microscopy	94	20 (10 – 35)	90 (78 – 97)	97 (93 – 99)	6 (5 – 7)	23 (15 -33)	TP 9 , FP 5<br>FN 36, TN 44
MPT64	107	100 (93 – 100)	2 (.05 – 10)	95 (95 - 95)	100 (3 – 100)	95 (89 – 98)	TP 52, FP 54<br>FN 0, TN 1
Xpert and/or MPT64	107	100 (93 - 100)	2 (.05 – 10)	95 (95 – 95)	100 (3 -100)	95 (89 – 98)	TP 52, FP 54<br>FN 0, TN 1
**Pediatric samples, CRS[e]**							
Xpert	10	60 (26-88)	-*	97 (50 – 100)	-	-	TP 6, FP 0<br>FN 4, TN 0
Culture	13	54 (25 – 81)	-*	96 (54 – 100)	-	-	TP 7, FP 0<br>FN 6, TN 0
ZN microscopy	36	17 (6 – 33)	-*	89 (41 – 100)	-	-	TP 6, FP 0<br>FN 30, TN 0
MPT64	41	95 (83 – 99)	0 (0 - 97)	98 (98 – 98)	-	93 (81 – 99)	TP 38, FP 1<br>FN 2, TN 0
Xpert and/or MPT64	41	95 (83 – 99)	0 (0 – 98)	98 (98 – 98)	-	93 (81 – 99)	TP 38, FP 1<br>FN 2, TN 0
**Pediatric, against culture**							
Xpert	6	50 (1 – 99)	25 (1 – 81)	97 (88 – 99)	1 (.1 – 8)	50 (12 – 88)	TP 1, FP 3<br>FN 1, TN 1
ZN microscopy	11	80 (28 – 99)	100 (54 – 100)	100 (40 – 100)	9 (2 – 37)	80 (47 – 97)	TP 4, FP 0<br>FN 1, TN 6
MPT64	13	100 (59 - 100)	17 (.4- 64)	98 (98 – 99)	100 (3 – 100)	98 (73 – 100)	TP 7, FP 5<br>FN 0, TN 1
Xpert and/or MPT64	13	100 (59 -100)	17 (.4 – 64)	98 (98 – 99)	100 (3 – 100)	98 (73 – 100)	TP 7, FP 5<br>FN 0, TN 1
**Adults, CRS [f]**							
Xpert	54	71 (56 - 83)	100 (29 - 100)	100 (90-100)	15 (10 - 21)	72 (58 -83)	TP 36, FP 0<br>FN 15, TN 3
Culture	95	48 (38 – 59)	100 (16 – 100)	100 (92 – 100)	9 (8 – 11)	51 (41 - 61)	TP 45, FP 0<br>FN 48, TN 2
ZN microscopy	264	11 (7-15)	100 (59 - 100)	100 (87 – 100)	6 (5 -6)	15 (11 – 20)	TP 27, FP 0<br>FN 230, TN 7
MPT64	310	94 (91 - 97)	82 (48 – 98)	99 (97 – 100)	43 (31 – 57)	94 (90 – 96)	TP 282, FP 2<br>FN 17, TN 9
Xpert and/or MPT64	312	94 (91- 97)	82 (48 - 98)	99 (97 – 100)	43 (31 – 57)	94 (90- 96)	TP 284, FP 2<br>FN 17, TN 9
**Adults against culture**							
Xpert	29	80 (28 - 99)	29 (13 - 51)	96 (93 – 97)	7 (1 -33)	77 (58 – 91)	TP 4 , FP 17<br>FN 1, TN 7
ZN microscopy	83	13 (4 - 27)	88 (74 – 96)	95 (86 – 98)	5 (4 – 6)	16 (9 – 26)	TP 5, FP 5

							FN 35, TN 38
MPT64	94	100 (92 – 100)	-**	95 (95 - 95)	-**	95 (88 – 98)	TP 45, FP 49<br>FN 0, TN 0
Xpert and/or MPT64	94	100 (92 – 100)	-**	95 (95 - 95)	-**	95 (88 - 98)	TP 45, FP 49<br>FN 0, TN 0

[a] Prevalence: 224/382 = 58%, [b] Prevalence: 22/57 = 39%, [c]Prevalence: 225/353 = 64%,
[d] Prevalence: 345/362 = 95%, [e] Prevalence: 41/42 = 98%, [f] Prevalence: 304/320 = 95%.

* No non-TB cases in these samples. ** no negative MPT64 results in these samples.

Abbreviations: CI; confidence interval, PPV; positive predictive value, NPV; negative predictive value, CRS; composite reference standard, ZN; Ziehl-Neelsen, TP; true positive, FP; false positive, FN; false negative, TN; true negative.

**Table S3. Positive MPT64 results and their correlation to cytomorphologic and histopathologic descriptions.**		
	**Positive MPT64 results in FNAC[a] samples**	
	**TB (n = 147)**	**Non-TB (n = 105)** [x]
All FNAC patterns considered TB	54/121 (45) [v]	15/33 (45)
**Gr. infl. w/o necrosis (TB (N = 9), non-TB: (N = 2))**	3 (33)	0 (-)
**Gr. infl. w/necrosis (TB: 88, non-TB: 15)**	33/82 (40)	9/14 (64)[c]
**Necrosis w/ few neutrophils (TB: 17, non-TB: 13)**	13 (76)	5/12 (42)[d]
**Caseous necrosis (TB: 13, non-TB: 5)**	5 (38)	1/5 (20)[e]
Reactive pattern (TB:17, non-TB: 54)	2 (12)	6/53 (11)
Other[b] (TB: 3, non-TB: 16)	1 (33)	1/15 (7)
	**Positive MPT64 results in biopsies**	
	**TB (n = 345)**	**Non-TB (n = 17)**
All histopathologic patterns considered TB	310/327 (95)	3/17 (18)
**Gr. infl. w/o necrosis (TB = 16, non-TB: 1)**	13/15 (87)	0 (-)
**Gr. infl. w/necrosis (TB = 308, non-TB = 3)**	289/303 (95)	2 (67)
**Necrosis only (TB = 9)**	8 (89)	-
Other [f] (TB = 12, non-TB = 13)	10 (83)	1/8 (13)

TB and non-TB groups according to CRS, n/N (%). Patterns suggestive of TB shown in bold.
[a] 2 cytology samples insufficient. [b] TB cases: Actinomycosis (*n* = 1), cystic lesion (*n* = 1), malignancy (*n* = 1), Non-TB cases: Acute abscess (*n* = 2), goiter (*n* = 4), malignancy (*n* = 6), benign tumor (*n* = 2), cystic lesion (*n* = 2). [c] Improved without ATT (*n* = 6), benign tumor (*n* = 1), other infections (*n* = 6), thyroiditis (*n* = 1), [d] acute abscess (*n* = 10), improved without ATT (*n* = 1), leprosy (*n*= 1).), [e] Improved without ATT (*n* = 2), other infections (n = 2) acute abscess (*n* = 1). [f] TB cases: Abscess (n = 12), Non-TB cases: Fungal infection (n = 8), Abscess (n = 3), Malignancy (n = 1), Urogenital inflammation (n = 1).

[v] 6 cases with insufficient MPT64 description. [x] 4 non-TB cases without MPT64 description.

NOTE: 13 samples had both a MPT64 result in cytology and biopsy sample. Among these samples three samples were MPT64 positive on both biopsies and cytology. One MPT64 positive cytology sample was negative on corresponding biopsy sample. Five additional cases were correctly identified as MPT64 positive on biopsies compared to cytology results.

Table S4. Diagnostic accuracy of cytology, routine TB diagnostics and the MPT64 test on FNA samples							
	n	Sensitivity (95% CI)	Specificity (95% CI)	PPV (95% CI)	NPV (95% CI)	Accuracy (95% CI)	
**All patients**[a]							
Cytology[b]	252	86 (80 – 91)	67 (57 – 76)	78 (73 – 83)	78 (70 – 85)	78 (72 – 83)	TP 127 FP 35 FN 20 TN 70
Xpert	254	34 (26 – 42)	100 (97 – 100)	100 (93 – 100)	52 (49 – 55)	62 (55 – 68)	TP 50 FP 0 FN 98TN 106
Culture	239	31 (23 – 39)	100 (96 – 100)	100 (92 – 100)	51 (48 – 54)	60 (53 – 66)	TP 42 FP 0 FN 95TN 102
ZN microscopy	254	19 (13 – 26)	99 (95 – 100)	97 (80 – 100)	47 (45 – 49)	53 (46 – 59)	TP 28 FP 1 FN 120 TN 105
MPT64	243	40 (32 – 49)	80 (71 – 87)	74 (64 – 81)	49 (45 – 53)	57 (50 – 63)	TP 57 FP 20 FN 85 TN 81
FNAC and/or MPT64	252	88 (82 – 93)	62 (52 – 71)	76 (71 – 80)	79 (71 – 86)	77 (72 – 82)	TP 130 FP 40 FN 17 TN 65
**Adults**[c]							
Cytology	205	87 (80 – 93)	62 (50 – 72)	79 (74 – 83)	75 (65 – 83)	78 (71 – 83)	TP 111 FP 30 FN 16 TN 48
Xpert	207	36 (28 – 45)	100 (95- 100)	100 (92 – 100)	49 (46 – 52)	60 (53 – 67)	TP 46 FP 0 FN 82 TN 79
Culture	195	33 (25 – 42)	100 (95 – 100)	100 (91 – 100)	48 (45 – 51)	58 (51 – 65)	TP 39 FP 0 FN 79 TN 77
ZN microscopy	207	21 (14 – 29)	99 (93 – 100)	96 (79 – 99)	43 (41 – 46)	51 (44 – 58)	TP 27 FP 1 FN 101 TN 78
MPT64	197	43 (34 – 52)	75 (63 – 84)	73 (64 – 81)	44 (39 – 49)	55 (48 – 62)	TP 52 FP 19 FN 70 TN 56
FNAC and/or MPT64	205	90 (83- 94)	55 (43 – 66)	77 (72 – 81)	77 (66 – 85)	77 (70 – 82)	TP 114 FP 35 FN 13 TN 43
**Pediatric cases**[d]							
Cytology	47	80 (56 – 94)	82 (62 – 94)	77 (59 – 88)	84 (69 – 93)	81 (67 – 91)	TP 16 FP 5 FN 4 TN 22
Xpert	47	20 (6 – 44)	100 (87 – 100)	100 (40 – 100)	62 (57 – 67)	66 (50 – 78)	TP 4 FP 0 FN 16 TN 27
Culture	44	16 (3 – 40)	100 (86 – 100)	100 (29 – 100)	61 (56 – 66)	64 (48 – 78)	TP 3 FP 0 FN 16 TN 25
ZN microscopy	47	5 (0.1 – 25)	100 (87 – 100)	100 (3 – 100)	58 (56 – 61)	59 (44 – 77)	TP 1 FP 0 FN 19 TN 27
MPT64	46	25 (9 – 49)	96 (80 – 100)	83 (38 – 97)	63 (57 -69)	66 (50 – 79)	TP 5 FP 1 FN 15 TN 25
FNAC and/or MPT64	47	80 (56 – 94)	82 (62 – 94)	77 (59 – 94)	84 (69 – 93)	81 (67 – 91)	TP16 FP 5 FN 4 TN 22

[a] Prevalence: 148/254 = 58%. [b] Cytology positive: granulomatous inflammation with or without necrosis, or necrosis without predominance of neutrophils or caseous necrosis [c] 128/207 = 62%, [d] 20/47 = 43%.

Abbreviations: CI; confidence interval, PPV; positive predictive value, NPV; negative predictive value, CRS; composite reference standard, ZN; Ziehl-Neelsen, TP; true positive, FP; false positive, FN; false negative, TN; true negative.

Table S5. Comparison of MPT64 diagnostic accuracy in biopsies from GDH, Pakistan and RDGMC, India.						
		**GDH (n = 329)**			**RDGMC (n = 43)**	
	**n**	**Sensitivity (95% CI)**	**Specificity (95% CI)**	**n**	**Sensitivity (95% CI)**	**Specificity (95% CI)**
Overall performance	310	95 (92 - 97)	83 (36 - 100)	41	86 (70 - 95)	67 (22 - 96)
**Site**						
Lymph nodes	240	97 (94 - 99)	-*	16	80 (52 – 96)	100 (3 – 100)
Pleural biopsies	34	93 (78 – 99)	75 (19 -99)	3	100 (29 -100)	-*
Other sites[a]	36	88 (73 – 97)	100 (16 – 100)	22	88 (64 – 99)	60 (15 – 95)

[a] GDH: Abscess (n = 24), Urogenital (n = 5), Skin (n = 2) Bone (n = 3) Gastrointestinal TB (n = 1), Pericardial (n = 1). RDGMC: Gastrointestinal (n = 5), Urogenital (n = 6), Bone (n = 5), Abscess (n = 2), Breast (n = 2), Skin (n = 1), Larynx (n = 1) , * no non-TB samples

Abbreviations: RDGMC: R.D. Gardi Medical College; GDH: Gulab Devi Hospital, CI; confidence interval.

Protocols for Immunohistochemical staining (IHC)

R.D. Gardi medical College

Formalin Fixed Biopsy

Alcohol-fixed smears: must be hydrated through decreasing grades of alcohol, start on rehydration step

- All incubations should be carried out at room temperature
- **Never let the slides dry during the staining procedure.** Dry tissue sections may display increased non-specific staining. If prolonged incubations are used, place tissues in a humid environment.

Control slides: (if biopsy controls are used, use IHC protocol for the biopsy slides and ICC protocol for smears)

- Negative control 1 : true positive sample – ONLY DILUENT
- Negative control 2 : true negative sample
- Positive control : true positive sample

**Deparaffinization step**		
1. Soak the slides in Xylene		5 min x 2
Soak the slides in Abs Alc.		5 min x 2
3. Soak the slides in 96%		5 min
4. Soak the slides in 70% alcohol	140 ml Abs. Alc. + 60 ml dd H_2O)	5 min
5. Distilled water		3 min x 2
**During re-hydration**<br>• Take the washing buffer (Tris buffer) out from the refrigerator<br>• Take the 'peroxidase' out from the refrigerator<br>• Take out the serum free protein block<br>• Dilute the primary antibody in Antibody Diluent solution: dilution 1:250 (for 1 ml: 4 µl primary antibody in 996 µl Antibody Diluent solution)<br>• Prepare the slide humidity chamber		
**Antigen retrieval step, Heat induced epitope retrieval (HIER)**		
Citrate buffer, pH 6.0	25 ml buffer + 225 ml dd H_2O<br>The boiling buffer can be used three times, remember to refill, to compensate for evaporation.	
**Microwave**<br>**Antigen retrieval solution (envision flex TRS 50x)+Distilled water in ratio 1:50**<br>Always place three containers in the microwave oven. Place slides and boiling buffer in one container and fill up the other two with water. This is to avoid evaporation from the container with buffer and slides.<br>**Bring buffer to boiling point, max power.**<br>**Slightly boiling for 15 minutes, 300-400 W**		
**Cooling**<br>Let container with boiling buffer come to room temperature, 20 min.		
**Washing step**<br>Move the rack with slides to a container with distilled water. 3 min.<br>TBS (Tris Buffered Saline) is used as wash buffer for manual staining.		

**Wash buffer bath**	Tris buffer pH 7,6 (TBS)	Use a Pasteur pipette and rinse the slides gently with wash buffer 3-4 times, then apply wash buffer to cover the specimen, after 5 min repeat the procedure. (Repeat 3 times)
**Dakopen**<br>**Carcinogenic**	Tap off excess buffer using a lint-less tissue, and carefully wipe around the specimen to remove any remaining liquid.	Encircle the region of tissue with the PAP pen to keep the reagents within the prescribed area

**Staining protocol**		
**Step 1: Peroxidase block**		
**Peroxidase block**	Apply enough Peroxidase Block to cover the specimen.	**30 min in slide humidity chamber**
**Wash buffer bath**	Tris buffer pH 7,6 (TBS)	Use a Pasteur pipette and rinse the slides gently with wash buffer 3-4 times, then apply wash buffer to cover the specimen, after **5 min** repeat the procedure. (Repeat 3 times)
**Step 2: Overnight incubation with mixture of 3% bovine serum albumin (BSA) and 10% normal goat serum (NGS)**		
	Apply enough solution to cover the specimen, place in refrigerator. Make sure that there is enough solution to avoid drying overnight	**Overnight in slide humidity chamber**
**NO WASH STEP. TAP OFF**		
**12 minutes with serum free block. No wash step, just tap off**		
**Step 3: Primary antibody with negative and positive control**		
**Primary antibody**<br>1:300 (IHC)<br>1:200 (ICC)	Tap off excess serum and wipe the slides as before.<br>Keep the reagents within the prescribed area<br>Apply enough optimally diluted primary antibody and negative control reagent (only antibody diluent)	**60 min in slide humidity chamber**
**Wash buffer bath**	Tris buffer pH 7,6 (TBS)	Use a Pasteur pipette and rinse the slides gently with wash buffer 3-4 times, then apply wash buffer to cover the specimen, after **5 min** repeat the procedure. (Repeat 3 times)
• Take the 'peroxidase labeled polymer' out from the refrigerator half an hour before the next step		
**Step 4: Peroxidase labeled polymer**		
**EnVision – labeled polymer HRP anti-rabbit**	Tap off excess water and wipe the slides as before.<br>Apply enough labeled polymer to cover the specimen.	**40 min in slide humidity chamber**
**Wash buffer bath**	Tris buffer pH 7,6 (TBS)	Use a Pasteur pipette and rinse the slides gently with wash buffer 3-4 times, then apply wash buffer to cover the specimen, after **5**

		**min** repeat the procedure.<br>(Repeat 3 times)
Take the AEC Chromogen out from the refrigerator and centrifuge 5 min. **Carcinogenic**		
**Step 5: AEC**		
**EnVision - AEC Chromogen**	Tap off excess water and wipe the slides as before.<br>Apply enough AEC chromogen to cover the specimen.<br>**Ventilation**	**15 min**<br>keep the reagents within the prescribed area
**Washing**	Rinse the slides gently with wash buffer (TBS) using a wash bottle or Pasteur pipette<br>**Collect substrate-chromogen waste in a hazardous materials container for proper disposal.**	Use a Pasteur pipette and rinse the slides gently with wash buffer 3-4 times, then apply wash buffer to cover the specimen, after 5 min repeat the procedure.<br>(Repeat 3 times)
**Distilled water**		4 min x 2, shaking
**Step 6: Hematoxylin counterstain** Always filtrate before use		
**Hematoxylin (Dako 3309)**	Immerse in aqueous hematoxylin bath	**30 seconds**<br>**(more or less)**
**Distilled water**	Slowly running tap water.<br>Place the slides in distilled water	**3 min**<br>**3 min**
**Step 6 : Mounting**		
**Cover with Immu-mount**	Tap of excess water, and wipe the slides as before. Let sections dry.<br>Put one or two drops of Mounting medium on the cover slip and gently place the cover slip over the slide<br>Get rid of the air bubbles trapped between the slides and the cover slip	

TBS (Tris Buffered Saline) AgilentDAKO: S3006
Antibody Diluent solution) AgilentDAKO: S0809
Protein Block, serum free AgilentDAKO: X0909
EnVision – labeled polymer HRP anti-rabbit AEC AgilentDAKO: K4009
Hematoxylin AgilentDAKO: S3309
Immu-mount mounting media: Shandon: 9990402

Immunocytochemistry (ICC) – R.D. Gardi Medical College

Slides for ICC were fixed in absolute ethanol for 1 hour and stored at -20^{0}C till further processed. For staining gradually got them at normal temperature.

For cocktail3 1:200 gave good result as compared to other dilution so this dilution were preferred for staining for slide in our study.

Staining procedure was performed as follow

1) slide were taken out of the refrigerator and brought to the normal temperature.

2) slide were kept in 80% alcohol for 4 min.

3) slide were kept in 70% alcohol for 4 min.

4) Slide were kept in distilled water for 10 min with gentle shaking

5) Slide were placed in humidifier chamber and rinsed with wash buffer for 3 times and cover the slide with wash buffer for 3 min, procedure was repeated for 3 times.

6) Excess of buffer was drained off and wipe with filter paper carefully.

7) smears were marked by Dako pen and peroxidase block was applied for 30 min.

8) Again slide were washed off using wash buffer same as step no 5.

9) Slide were gently wiped and primary antibody was applied for 1 hour.

10) slide were washed as in step no 5 then secondary antibody rabbit anti HRP was applied for 50 min.

11) wash the slide using wash buffer as in step no 5 then apply AEC chromogen for 15 min.

12) wash the slide using wash buffer as in step no 5 and then kept the slide in distilled water for 10 minutes with gentle shaking.

13) slides were counter stained with Mayer’s hematoxylin.

14) slides were blued in tap water for 3 min

15) slides were kept in distilled water for 3 min.

16) after drying of slide mounted with DPX.

Throughout the procedure the slides were kept moist. Each staining run included a positive and negative control.

MPT64 Staining Protocol (IHC)

Gulab Devi Hospital

Formalin Fixed Biopsy:

Alcohol fixed smears: must be hydrated through decreasing grades of alcohol, start on rehydration step.

All incubations should be carried out at room temperature.

Never let the slides dry during the staining procedure. Dry tissue sections may display increased nonspecific staining. If prolonged incubations are used, place tissues in a humid environment.

Control slides:

Negative control 1: True positive sample (Only Diluent)

Negative control 2: True negative sample

Positive control: True positive sample

Deparaffinization step:

1. Soak the slides in xylene. 5 min x 2
2. Soak the slides absolute Alc. 5 min x 2
3. Soak the slides in 96%. 5 min
4. Soak the slides in 70% alcohol. 5 min
   (140 ml Abs. Alc + 60 ml add H_2O)
5. Distilled water 3 min x 2

During re-hydration:

- Take the washing buffer (Tris buffer) out from the refrigerator.
- Take the 'peroxidase' out from the refrigerator.
- Take out the serum free protein block.
- Dilute the primary antibody in Antibody. Diluent solution dilution 1:250 (for 1 ml: 4 ml primary antibody in 996 µl antibody diluent solution)
- Prepare the slide humidity chamber.

Antigen Retrieval step, Heat induced epitope retrieval (HIER)

Citrate buffer, pH 6.0 = 25 ml buffer + 225 ml distil H_2O

The boiling buffer can be used three times, remember to refill to compensate for evaporation.

Microwave:

Always place three containers in the microwave oven. Place slides boiling buffer in one container and fill up the other two with water. This is to ovoid evaporation from the container with buffer and slides.

Bring buffer to boiling point, max power. Slightly boiling for 15 minutes, 300-400W.

Cooling:

Let the container with boiling buffer come to room temperature, 20 min.

Washing step:

Move the rack with slides to a container with distilled water 3 min.

TBS (Tris Buffered Saline) is used as wash buffer for manual staining.

Wash buffer bath	Tris buffer pH 7.6 (TBS)	Used a Pasteur pipette and raise the slides generally with was buffer to cover the specimen, after 3 min repeat the procedure. (Repeat
Dako pen (Carcinogenic)	Tap offers excess buffer using lint less tissue and carefully wipe around the specimen to remove any remaining liquid.	Encircle the region of tissue with PAP pen to keep the reagents within the prescribed area.

Staining Protocol:

Step 1: Peroxidase block:

Peroxidase block	Apply enough peroxidase block to cover the specimen	20 min in slide humidity chamber
Wash buffer bath	Tris buffer pH 7.6 (TBS)	Use a pasteur pipette and rinse the slides gently with wash buffer 3-4 times, then apply wash buffer to cover the specimen, after 3 min repeat the procedure (Repeat 3 times)

Step 2: Overnight incubation with mixture of 3% bovine serum albumin (BSA) and 10% normal goat serum (NGS)

	Apply enough solution to cover the specimen, place in refrigerator make sure that there is enough solution to ovoid drying overnight	Overnight in slide humidity chamber

NO WASH STEP TAP OFF

For some slides an extra step of 12 min with serum free block. No was step just tap off.

Step 3: Primary antibody with negative and positive control.

Primary antibody<br><br>1: 250 dilution	Tap off excess serum and wipe the slides as before. Keep the reagents within the prescribed area. Apply enough optimally diluted primary Ab and negative control reagent. (Only antibody diluent)	60 min in slide humidity chamber

Wash buffer bath	Tris buffer pH 7.6 (TBS)	Use a Pasteur pipette and rise the slides gently with wash buffer 3-4 times, then apply wash buffer to cover the specimen after 3 min repeat the procedure (Repeat 3 times)

- **Take the 'peroxidase labeled polymer' out from the refrigerator half an hour before the next step.**

Step 4: Peroxidase labeled polymer:

En vision<br><br>FLEX/HEP anti rabbit	Tap of excess water and wipe the slides as before. Apply enough labeled polymer to cover the specimen	40 minimum slides in humidity chamber
Wash buffer bath	Tris buffer pH 7.6 (TBS)	Use a Pasteur pipette and rinse the slides gently with wash buffer to cover the specimen after 3 min repeat the procedure (Repeat 3 times)

Take the 'DAB chromogen' out from the refrigerator and centrifuge 5 min. Carcinogenic.

Step 5: DAB

Envision-DAB chromogen	Tap of excess water and wipe the slides as before. Apply enough DAB chromogen to cover the specimen.<br><br>Ventilation	15 min keep the reagents with the prescribed area.
Washing	Rinse the slides gently with wash buffer (TBS) using a wash bottle or Pasteur pipette.<br><br>Collect substrate-chromogen waste in a hazardous materials containers for proper disposal.	Use a Pasteur pipette and rinse the slides gently with wash buffer 3-4 times, then apply wash buffer to cover the specimen after 3 min repeat the procedure (Repeat 3 times)
Distilled water		4 min x 2<br>Shaking

Step 6: Hematoxylin counterstain:

Always filtrate before use.

Haris Hematoxylin (Merck)	Immerse in aqueous Hematoxylin bath	30 seconds (more or less)
Distilled water	Slowly running tap water	3 min

	place the slides in distilled water	3 min

Step 7: Mounting

Cover with DPX	Tap of excess H_2O and wipe the slides as before the slides as before. Let sections dry. Put one or two drops of mounting medium on the cover slip and gently place the cover slip over the slide. Get rid of the air bubbles trapped between slides and the cover slip.
